# Skeletal muscle TFEB overexpression does not increase neurogenesis markers in the young female hippocampus

**DOI:** 10.17912/micropub.biology.001612

**Published:** 2025-05-06

**Authors:** Mia Hakian, Ian Matthews, Constanza J. Cortes

**Affiliations:** 1 Leonard Davis School of Gerontology, University of Southern California, Los Angeles, California, United States

## Abstract

Adult hippocampal neurogenesis (AHN), the process in which new neurons are formed in the dentate gyrus of the hippocampus, declines with age and is highly responsive to voluntary wheel running in mice. This exercise-activated increase in AHN is believed to contribute to the cognitive and neurotrophic benefits of exercise on the aging and neurodegenerative disease-afflicted brain. However, our current understanding of the decline in AHN remains male-centric, with very few studies examining the effects of age and/or running on AHN in the female brain. Our lab has recently shown that skeletal muscle-specific overexpression of Transcription Factor E-B (TFEB), a master regulator of lysosomal and mitochondrial function, mimics many of the neuroprotective benefits of exercise during aging and in the context of Alzheimer’s disease (AD) pathologies, but the effect of muscle-TFEB overexpression on AHN was unknown. Here we report that female AHN declines in a similar timeline as to what has been reported for the male hippocampus, following a precipitous decline at around 3 months of age that culminates at around 8 months of age. Furthermore, we report that muscle-TFEB overexpression does not prevent this age-associated decrease in AHN, suggesting that the neuroprotective benefits observed in our muscle-TFEB model are independent of AHN.

**Figure 1. Exponential age-associated decline in the female neurogenesis is not altered by overexpression of TFEB in skeletal muscle:**
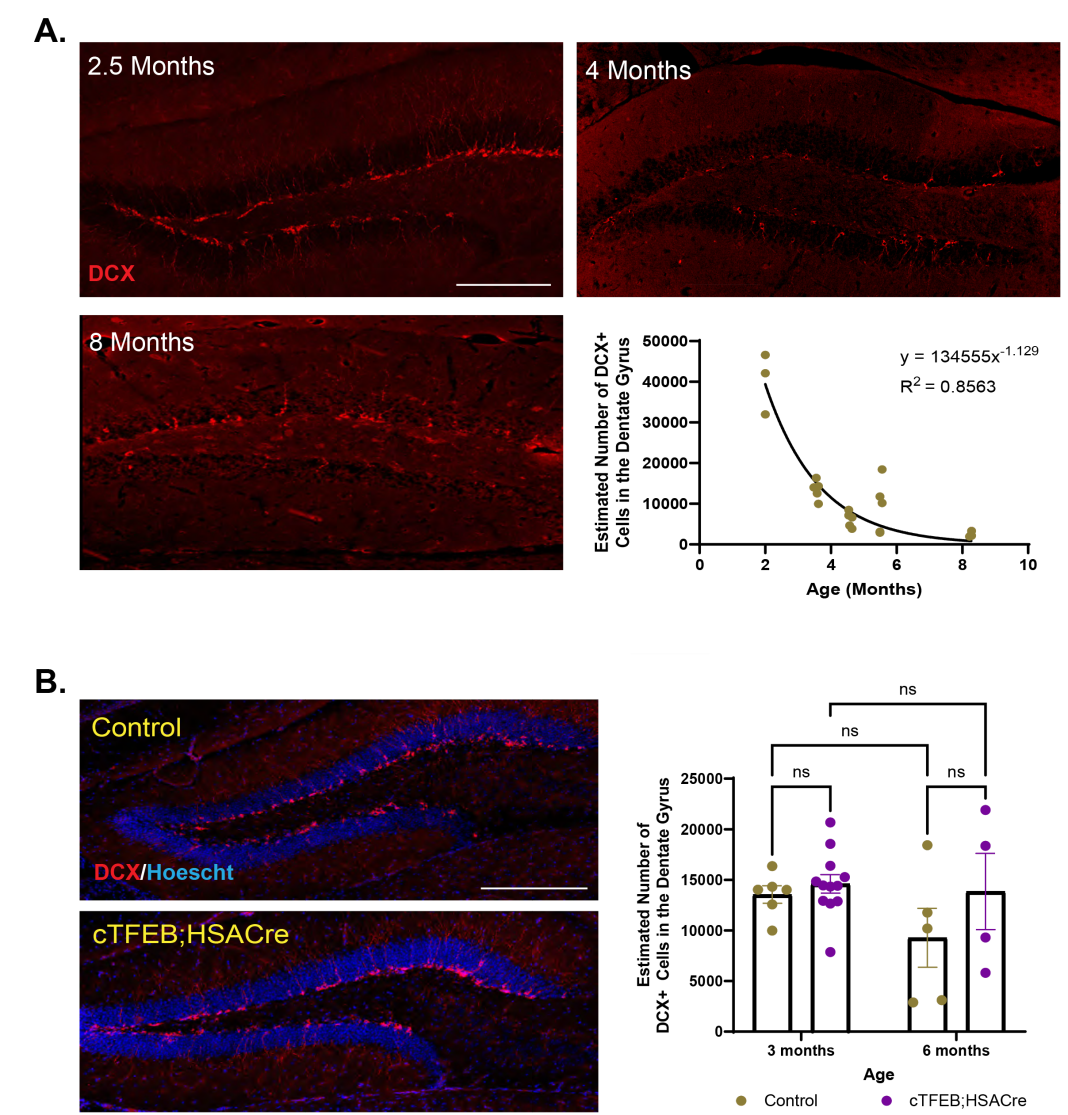
**(A)** Representative images of the dentate gyrus of the control hippocampus at 2.5, 4 and 8 months of age, and quantification of estimated number of DCX+ cells across different age time points. Best fit exponential line is shown. **(B)** Representative images of the dentate gyrus of control (top) and cTFEB;HSACre (bottom) transgenic mice at 3 months of age. Quantification of estimated DCX+ cells across age-matched littermate groups is shown on the right. Doublecortin (DCX) stain in red, Hoescht nuclear stain in blue. Scale bar = 100 µm. Data is presented as total numbers (A) or mean ± SEM (B). Two-way ANOVA with post-hoc multiple comparisons, n.s.: non-significant

## Description

Neurogenesis is the process through which neural stem cells give rise to new neurons in the murine hippocampus, and it is highly activated by running ^1^ . Conversely, an exponential decline in adult hippocampal neurogenesis (AHN) is observed during aging, with the number of neural stem cells and their newly born neurons precipitously declining shortly after mice achieve sexual maturity (~3-8 months of age) ^2,3^ . To date, most research into exercise-activated neurogenesis has been focused on only one sex (males) and few studies have examined whether sex contributes as a biological variable to this age-associated decline in AHN ^4^ .

We have recently demonstrated that overexpression of Transcription Factor E-B (TFEB) in skeletal muscle (cTFEB;HSACre transgenic model) is sufficient to drive multiple exercise-like benefits in the CNS ^5,6^ . Indeed, we have confirmed neuro- and geroprotective effects in the hippocampus of cTFEB;HSACre in the absence of any exercise activity, including reductions in neuroinflammation, increases in neurotrophic signaling and reductions in tau- ^5^ and amyloid beta-associated pathologies ^6^ . Interestingly, these neuroprotective effects appear to be sex-dimorphic, suggesting that muscle-TFEB and/or exercise may drive CNS resilience via functionally convergent mechanisms in either sex ^7^ . However, to date, we have not examined whether muscle-TFEB overexpression can also modify the age-associated trajectories of AHN decline.

To assess AHN in this context, we quantified the number of doublecortin (DCX) positive cells in the dentate gyrus of the hippocampus via immunofluorescence in mouse hemibrain cryosections. DCX labels immature neurons and has been extensively utilized as a marker for neurogenesis during development and in paradigms known to increase neurogenesis levels, including exercise ^3,8-10^ . To begin our analysis, we first assessed the number of DCX-positive neurons in the hippocampus of female control mice at multiple time points. Consistent with what has been previously reported in similar studies in male mice ^4,11,12^ , we found an exponential decline in the estimated number of DCX+ cells in the female hippocampus across aging ( **Figure 1A** ). At 2.5 months of age, a time of peak AHN activity associated with brain maturation and development, we estimated 40240 ∓ 4329 DCX+ neurons to exist in the dentate gyrus of the female hippocampus. At 3.5 months of age, just a month later, this number had sharply decreased to 13547 ∓ 866 DCX+ cells in the same brain region. This trend continued at 4.5 months (6150 ∓ 845.5 cells) and 5.5 months (7730 ∓ 2835 cells) of age. By 8 months of age, this number had dropped to only 2410 ∓ 311 DCX+ cells, consistent with existing reports suggesting that mouse dentate gyrus AHN decreases 15-20 fold by 9-12 months of age relative to neonatal levels ^13,14^ .

It is generally understood that AHN undergoes an exponential decline of neurogenic parameters across age ^11-13^ . We mapped the distribution of DCX+ positive cells in the female hippocampus across all our age time points and determined the best fit to be a non-linear exponential distribution (R ^2^ = 0.8563), which parallels what has previously been reported for the male mouse hippocampus ^3^ . It is important to note that the population of DCX-labeled cells might not provide a complete estimate of the number of functional new neurons, as only a fraction of DCX-labeled cells will survive to become fully functional. However, our results suggest that, similar to what is known about the male hippocampus, female AHN proceeds in a similar trajectory, peaking at 2-3 months of age and rapidly declining to negligible numbers by 9 months of age.

With this in mind, we evaluated AHN in the context of our newly developed exercise-mimetic model, the cTFEB;HSACre transgenic mice ^5^ . We chose a first age time point with dynamic levels of AHN (3.5 months of age) and a second time point where AHN has significantly declined (5.5 months of age) to determine whether muscle-TFEB overexpression can modify AHN in the female hippocampus **( Figure 1B** ). Confirmation of TFEB overexpression in the skeletal muscle of these same cohorts has been previously published in our original work describing the CNS targeting benefits of muscle-TFEB overexpression ^5^ . We found that there was no significant difference in the estimated number of DCX+ neurons in the hippocampus of female control and cTFEB;HSACre transgenic mice at 3 months (average of 13547 ∓ 866 vs. 14605 ∓ 917.4 cells, respectively) or 6 months (average of 9276 ∓ 2911 vs. 13845 ∓ 3767 cells respectively) of age. Although our limited number of time points in this cohort prevented us from examining AHN trajectories as done in Figure 1A, this data suggests that muscle-TFEB overexpression does not increase the number of DCX+ neurons in a highly neurogenic environment (3 months old), nor does it protect against age-associated decline of AHN (6 months old) ( **Figure 1B** ).

These results were surprising to us given our published results highlighting multiple neurotrophic, exercise-like effects of muscle-TFEB overexpression on the hippocampus ^5,6^ . This indicates that the neuroprotective effects of muscle-TFEB overexpression, including improved neurocognitive performance, reduced neuroinflammation, and increased neurotrophic signaling ^5,6^ are probably independent of AHN reactivation. This disparity also suggests that AHN activation is not required for the full manifestation of the neuroprotective benefits associated with exercise, an intriguing possibility with high therapeutic potential for aging populations where AHN may have reached the ‘point of no return’.

## Methods

*Animals.* We have previously described the generation of fxSTOP-TFEB transgenic mice ^5^ . For this work we utilized non-transgenic mice (~2-9 months of age) or cTFEB;HSACre double transgenic female mice and their non-transgenic or single transgenic littermate controls (3-6 months of age). In our hands, we have not detected any differences in CNS phenotypes on fxSTOP-TFEB+ or HSA-Cre+ single transgenic animals compared to the wild-type littermates ^5^ , so they are all combined into our ‘control’ group (Figure 2).

*Tissue collection.* Animals were anesthetized with 3.8% Avertin Solution prior to tissue collection. All animals received a transcardial perfusion with 60 mLs of cold 1x PBS. Half of the brain (right/left hemi-brain) was post-fixed in 4% PFA for less than 24 h before being transferred to 30% sucrose for another 24 h before cryo-sectioning and staining.

*Immunofluorescence Analysis.* Hemibrains were embedded in OCT (TissueTek, 4583), frozen utilizing 2-methylbutane (VWR, 103525-278) vapor phase submerged in liquid nitrogen, and stored at 80 °C until used. All samples were sectioned on a standing cryostat (Leica). Brain sections were 20 µm thick. For immunofluorescence staining of doublecortin (DCX), antigen retrieval was performed using 1x citrate buffer in a high-pressure cooker for 6 minutes on high followed by a quick release step. After cooling down for 10 minutes, slides were washed with 0.1% Triton for 15 minutes, then again with 1x PBS. Brain sections were then blocked with 4% BSA for 1 hour, followed by staining with primary doublecortin antibody #4604 (Cell Signaling, 4604S) (1:200) overnight at 4ºC and finally incubated with Donkey anti-rabbit Alex fluor 555 secondary antibody at RT for 1 h (both diluted in 4% BSA). Slides were washed with PBS-Hoescht (ThermoFisher, 62249, 1:5000) and mounted with Prolong Glass Antifade Mountant (Invitrogen, P36984). All slides were washed with 1X PBS three times for 5 min each between steps. All slides were imaged with an ECHO Revolution epifluorescent microscope using the TXRED and DAPI channels. Estimation of total DCX+ numbers per individual was determined by design-based stereology for standardized quantification of adult neurogenesis ^15^ , with minor modifications. In short, we used raw counts from 2-dimensional representative brain sections from each individual to estimate 3-dimensional populations in the entire hippocampus. In our hands, the entire cross-length of the adult mouse hippocampus was contained within 120 sections of 20 µm thickness, for an average length of 2.4 mm, consistent with information provided by the Allen Brain Atlas. After establishing our size parameters, we quantified every 16 ^th^ section of tissue for every individual, to cover the proximal, medial and anterior dentate gyrus. Quantification of raw DCX+ cells was performed in FIJI/ImageJ using the cell counter plug-in, number of sections sampled was consistent within groups. We determined the estimated number of DCX+ cells in the entire hippocampus via volumetric extrapolation. Our estimated DCX+ numbers are within range of previous publications utilizing a similar approach ^15^ .

*Statistical Analysis.* 2-way ANOVA and post-hoc multiple comparisons, as well as best-fit nonlinear regression curves were used to determine significance of differences in Prism 10.4.2 (GraphPad Software). P < 0.05 was considered statistically significant.
